# Exploring smoke: an ethnographic study of air pollution in rural Malawi

**DOI:** 10.1136/bmjgh-2021-004970

**Published:** 2021-06-30

**Authors:** Sepeedeh Saleh, Henry Sambakunsi, Kevin Mortimer, Ben Morton, Moses Kumwenda, Jamie Rylance, Martha Chinouya

**Affiliations:** 1 Liverpool School of Tropical Medicine, Liverpool, UK; 2 Malawi-Liverpool-Wellcome Trust Clinical Research Programme, Blantyre, Malawi

**Keywords:** public Health, environmental health, epidemiology, prevention strategies, qualitative study

## Abstract

Air pollution adversely affects human health, and the climate crisis intensifies the global imperative for action. Low-/middle-income countries (LMIC) suffer particularly high attributable disease burdens. In rural low-resource settings, these are linked to cooking using biomass. Proposed biomedical solutions to air pollution typically involve ‘improved cooking technologies’, often introduced by high-income country research teams. This ethnography, set in a rural Malawian village, aimed to understand air pollution within its social and environmental context. The results provide a multifaceted account through immersive participant observations with concurrent air quality monitoring, interviews and participatory workshops. Data included quantitative measures of individuals’ air pollution exposures paired with activity, qualitative insights into how smoke is experienced in daily life throughout the village, and participants’ reflections on potential cleaner air solutions. Individual air quality monitoring demonstrated that particulate levels frequently exceeded upper limits recommended by the WHO, even in the absence of identified sources of biomass burning. Ethnographic findings revealed the overwhelming impact of economic scarcity on individual air pollution exposures. Scarcity affected air pollution exposures through three pathways: daily hardship, limitation and precarity. We use the theory of structural violence, as described by Paul Farmer, and the concept of slow violence to interrogate the origins of this scarcity and global inequality. We draw on the ethnographic findings to critically consider sustainable approaches to cleaner air, without re-enacting existing systemic inequities.

**Key questions**

**What is already known?**

- Air pollution is a leading cause of global morbidity and mortality, and an important driver of health inequalities. Traditional global health approaches typically use individualised ‘cleaner cooking’ interventions, with limited successes in reducing cooking-related emissions in low/middle-income countries settings. Sustainable, clinically important improvements in health outcomes have been more challenging to achieve.

**What are the new findings?**

- Air pollution exposures in rural Malawi exceeded internationally recommended maximum levels even in the absence of identified sources of burning. Compounding this high background, intense exposures were identified during cooking, which constituted the greatest single identifiable contributor to poor air quality.
- Ethnographic findings demonstrate the striking impacts of economic scarcity on air pollution, and on communities’ capacities to avert their exposures.

**What do the new findings imply?**

- Air pollution does not exist in isolation: it is part of a wider environment which structurally compromises respiratory health. Effective interventions to improve lung health must be context informed and engage with communities’ lived experiences. A ‘geographically broad’ and ‘historically deep’ analysis of health determinants is invaluable to global health enquiry.

## Introduction

Evidence of the direct and indirect health effects of air pollution is overwhelming, as is recognition of its role in the escalating climate crisis.^1–4^ In Malawi, studies have demonstrated high domestic levels of harmful airborne particulates.^5 6^ Acute respiratory infection and chronic lung disease are common in this population. These are associated with poor air quality and share other causative factors such as poverty and malnutrition.^7–9^ Cooking using biomass (organic matter used as fuel) is known to be important, although other sources of air pollution are also present.^10–14^

With over 3 billion people worldwide relying on polluting fuels and technologies for cooking, these issues are key to global health.^15^ Evidence for the effectiveness of improved cooking technologies and clean fuels is mixed. Many interventions are insufficient to reduce particulate levels to below internationally recommended thresholds, or to improve health outcomes.^16–18^ In Malawi, a well-powered trial of efficient fan-assisted biomass stoves did not significantly reduce pneumonia in children.^19^ The exact relationship between exposure reduction and clinical outcomes is unclear, particularly for modest reductions in particulates. Nevertheless, there is widespread promotion of more basic biomass stoves. Advocates cite their wider benefits to the environment, and potential to support livelihoods through local manufacture.^20^

Suboptimal reductions in exposure in interventional trials have been linked to behavioural factors such as non-exclusive and poorly sustained use of the new technologies and fuels.^21–23^ Additional pollution sources include concurrent use of traditional cooking methods (‘stacking’), or non-cooking-related biomass combustion.^13 24^ Combining a quantitative assessment of air quality with an understanding of individuals’ cooking related concerns and motivations could contribute to improving outcomes.

Research on enablers and barriers to the adoption and sustained use of cleaner cooking fuels and technologies reveals various interacting factors.^25–28^ Lack of affordability and access prevent the uptake of new cooking technologies and fuels in many settings.^29–34^ Some studies suggest that health concerns can motivate transition to cleaner cooking technologies, but knowledge of the health harms of smoke does not necessarily lead to improved stove uptake and use.^29 35^ Both enabling and limiting factors are shaped by structural context (eg, clean energy availability), and cultural and social aspects.^24 27 28 31 36 37^ A study of four neighbouring Southern African countries (South Africa, Mozambique, Malawi and Zambia) revealed differing priorities, despite similarities in how individuals valued fuel and cost savings.^38^

Cookstove development approaches have changed over time. Analysis by Sesan^39^ regards this as transition from an ‘expert based’ position through to ‘market based’ approaches, noting that agendas are framed by high-income country actors throughout, often in response to their shifting priorities (initially health and subsequently climate). While analyses tend to focus on interventions, the author cites evidence of basic, more immediate needs competing for very limited resources in many settings. It is suggested that the ‘local’ population be engaged as active agents in the process, choosing priorities rather than acting as passive recipients.^39^

We used ethnography—including immersive participant observation in the village context—to bring alternative perspectives. We sought to understand individuals’ daily realities rather than starting with proposed solutions, and to bring together participants’ own knowledge developed through lived experience with our knowledge as clinicians and academics. The anthropologist João Biehl articulates this as, ‘rejecting the division between those who know the world and those who must simply struggle to survive it’ (40, p135).

The aim was to provide an account of air pollution in the context of the wider hardships, risks and limitations inherent to life in this setting. Our theoretical analysis incorporates the concept of structural violence, which describes how structures such as political, legal and economic systems can limit individuals, preventing them from reaching their full potential.^41^ This can include limiting of access to basic needs such as water, food and agency, as well as education and healthcare.^42^ Our critical analysis demonstrates how the context of global economic inequity can dominate individual lives and air pollution exposures in rural Malawi.^43 44^ This informs recommendations on meaningful and equitable approaches to air quality and broader environmental issues.

## Methods

### Study setting

The study, starting in June 2020, focused around a village of approximately 300 households (1800 individuals) on the outskirts of Blantyre, Malawi’s commercial capital. The village itself is rural, in common with 83% of the country’s population.^45^ Residents speak mainly Chichewa, the most widely spoken language nationally, with limited levels of spoken English. Households include men and women of all ages, with extended families frequently living in household clusters. There are many female-headed households, as men seek employment in urban areas or neighbouring countries. Economic insecurity is common, with income predominantly derived from ad hoc piecework or self-employment.^46 47^ The widespread use of solid fuel—mostly wood—for cooking, and a communal pump for water in the village reflect ways of life typical across rural Malawi.^48^ Deforestation is increasing nationally, related predominantly to wood use for cooking and to farmland clearing.^49^

### Historical and global context informing the ethnography

Malawi’s current economic situation, and wider transnational inequities, stem from five key global dynamics (box 1).^50^

Box 1 **Five key points of origin of global inequity**

1. Colonial processes
2. Colonial influences on postcolonial regimes in newly independent nations
3. Structural adjustment programmes
4. Recent international systems of trade
5. Global climate inequity

Malawi, as Nyasaland, was under British control from 1891 until independence in 1964. This period was characterised by extractive agricultural practices in which colonially appropriated land was distributed to European settlers for growing export crops. Resident Malawians were exploited for agricultural labour (the ‘thangata’ system), and hut and poll taxes introduced to move Malawians into the labour market.^51^ After independence, Malawi was reliant on crop exports and labour emigration, firmly establishing structural poverty.^52^ Today, Malawi remains dependent on extractive modes of trade through export of raw materials for processing and manufacturing elsewhere.^53^

Structural adjustment in 1981, precipitated by the oil crisis of the 1970s, brought financial assistance from the World Bank, conditional on extensive policy reforms.^54 55^ Trade liberalisation and deregulation opened countries such as Malawi to aggressive foreign markets with highly subsidised agriculture.^56^ Enforced privatisation of national assets undermined democracy, harmed health, and removed social protection systems, particularly impacting vulnerable groups.^54 56–58^ In Malawi for example, forced economic restructuring to repay high-interest loans precipitated a famine which caused hundreds of deaths in 2001–2002.^59 60^

Recent droughts and floods, intensified by climate crisis, add to the daily challenges of rural life. Widespread deforestation amplifies the damage in flood-hit areas. These situations are characterised by large-scale inequity. Globally, the richest 10% of the population are responsible for 52% of recent carbon emissions, while the poorest half generate only 7%.^61 62^ Food and economic insecurity are dominant issues in a population where smallholder farming accounts for 80% of food needs and where 38% live below the poverty line.^63 64^

### Study design and approaches

The research was devised and led by a doctoral researcher of British background (SS), based at a UK institution. The core study team also included a Malawian research assistant, and Malawian fieldworker resident in the village. Supervisory staff were of Southern African and British backgrounds.

**Figure 1:**
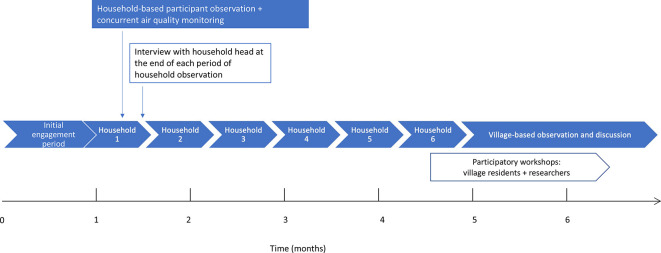
Combination of methods over the 7-month ethnographic period (further detail provided in online supplemental materials).

The study used a basis of in-person participant observation around the village over 7 months, with additional research methods superimposed throughout this period. Data were brought together at the analysis stage. This allowed for a more rounded understanding of the issue than could be gained through air quality monitoring, interviews, or participant observation research alone. Multiple methods allowed assessment of the consistency of the findings across different methods (‘triangulation’), contributing to the credibility dimension of ‘trustworthiness’ in qualitative research.^65 66^ The sequence of methods is shown in figure 1. Related discussions of research approaches, participant contributions, and ownership of the research product may be found in the online supplemental materials.

10.1136/bmjgh-2021-004970.supp1 Supplementary data

### Quantitative methods

Individual air quality monitoring was conducted during a sample of focused household participant observation periods (described below), measuring levels of inhaled fine particulate matter (PM_2.5_) at 2 min intervals. Monitoring in each household started only after a period of routine participant observation; monitoring periods were based on participants’ convenience and acceptability, with purposive sampling approaches ensuring a variety of household types, cooking factors and additional combustion sources were included.

Researchers wore the monitors while taking part in cooking and other activities alongside key household members. A subgroup of participants continued carrying monitors overnight after researchers left the household and were asked the following morning to identify key potential exposures. Extended monitoring incorporating a larger, more representative sample; repeated 24-hour monitoring periods; and spanning multiple seasons was also carried out. These datasets are currently under analysis and will be published separately.

PurpleAir PA-II laser particle counting devices (PurpleAir, Utah, USA), were used for PM_2.5_ monitoring, connected to 20 000 mAh portable power banks (Anker Innovations, Changsha, China). These low-cost monitors show excellent correlation with reference standard gravimetric analysis (GRIMM reference method; R^2^=0.98), with field use in various African settings.^67 68^ Monitors and power banks were carried in specially designed waist bags which held the device but did not cover any the intake (sampling) port.

Each trace was partitioned into ‘activity’ (usually cooking) and ‘baseline’ periods using paired activity data (from ethnographic records and participant reports). These data were analysed using STATA V.15.1 (StataCorp), calculating time weighted median exposures at baseline and during identified activity periods. We assessed the association of cooking features and location against PM_2.5_ using multilevel mixed effects linear regression analysis. The fixed effect was mean PM_2.5_ level and random effect was participant identifier (data and code available online: https://doi.org/10.7910/DVN/YPGUEH). Extended analyses from a broader dataset are being prepared for separate publication.

### Qualitative methods

Following gradual community introduction and consent processes, we undertook six periods of focused household participant observation, each lasting 3–4 weeks. This was followed by participant observation in various sites around the village, allowing access to a wider range of residents, sites, and activities. Researchers (SS, accompanied by the research assistant and/or local fieldworker) carried out routine activities alongside residents, including cooking, farming and visits to the local market, with ad hoc conversations providing opportunities for deeper exploration of specific issues. Notes around smoke exposure and wider aspects of daily life were taken contemporaneously. During later conversations with village members, early themes were raised for discussion, bringing participants’ perspectives into the analysis.

Individual interviews were held with household heads at the end of each household observation period, helping to confirm and clarify key findings. Finally, six once-weekly participatory workshops took place alongside the final weeks of participant observation, involving existing and new participants from the village, and researchers. These workshops, led in Chichewa by an external facilitator, used theatre-based participatory methods to challenge traditional power dynamics often inherent in transnational research projects.^69 70^ These methods are explained in further detail in the online supplemental materials. Early workshops explored the roles of ‘smoke’ in residents’ lives and later sessions encouraged participants to collectively consider ways of reducing exposure.

Interviews and most workshops were audio recorded, translated and transcribed throughout the fieldwork. Transcripts and fieldnotes were entered onto QSR NVivo V.12 software and independently coded by SS and HS, who then worked together on developing and refining themes from the data. This integration of different perspectives added to the credibility of the analysis.^65 71^ Early findings were used to iteratively focus the ongoing fieldwork.^72^ The theories we cite were arrived at through our findings in the village and were not predetermined.^73^

### Ethical considerations

The study was approved and sponsored by the LSTM Research Ethics Committee (19-007). In-country ethical approval was granted by the College of Medicine Research Ethics Committee (COMREC) in Blantyre (P.02/19/2600). Ethical enquiry, running through the project, was characterised by the ‘relational ethics’ approach^74^ as previously described.^75^

### Patient and public involvement

The local community guided methodological decisions throughout the ethnography. The involvement of a resident ‘village fieldworker’ enhanced the integration of community perspectives into the study. We discussed developing findings with residents throughout, particularly during individual and small group discussions. Participants’ contributions to analysis are discussed further in online supplemental materials. At the end of the participant observation period, key results were disseminated across the village using simple leaflets, and at a meeting with a group of key stakeholders from the village.

## Results

### Air pollution: levels and sources

Air quality data incorporated 203 monitoring hours and over 6104 datapoints,^76^ including 31 female and 14 male participants. PM_2.5_ concentration demonstrated a ‘baseline and peaks’ pattern, with spikes corresponding to specific exposure sources such as cooking when analysed in parallel with observational data (see representative trace online supplemental figure S1). Air quality monitoring results, coupled with information from interviews and workshops, revealed cooking to be the most important source of airborne particulate exposure, both in terms of frequency and magnitude.

Approximately 31% of the pooled traces were composed of ‘activity’ (mainly cooking). In the other 69% of time, with no identified biomass burning, median PM_2.5_ was 35.2μg/m^3^ (WHO recommendation: 24-hour average less than 25μg/m^3^).^4 77^ Intense peaks of particulate matter exposure were predominantly related to cooking, with the highest levels associated with open-fire cooking and cooking in poorly ventilated areas such as kitchens (online supplemental table S2). Levels peaked over 1000 μg/m^3^ in 29 of the 31 female traces (all cooking related). These activities typically took place three times a day, lasting 45–60 min. The median cooking activity-related exposure level across the study was 386 μg/m^3^. Cooking was exclusively done by women, often assisted by children (mainly female), and frequently with infants carried on their mothers’ backs as they cooked. Additional exposures for women included the warming of bathwater and occasional home-based business ventures such as roasting nuts or distilling alcohol.

More distant exposure sources were noted during observations including fires in neighbouring compounds or village brick-ovens, although concurrent personal exposure monitoring did not reveal perceptible peaks during these periods. Other particulate sources are summarised in table 1.

**Table 1:** Non-cooking related sources of exposure to airborne fine particulate matter (PM_2.5_) in and around the village

Activity	Population group exposed	Frequency/duration
Brick ovens: stacks of clay bricks fired in the open using wood combustion	Mainly men, who gained income from brick making	Twice per year on average, burning continuing for approximately 48 hours
Burning of farmland	Any residents close to sites of burning (although individuals rarely continued working on the farm after burning was started and so these exposures were not captured on traces)	Sporadic through the dry season. Observations and participant accounts noted fires typically burning for short periods of time—often less than 10 min—although ‘smouldering’ may have continued after this time
Visits to the roadside market (roads lined with idling motor vehicles)	Village residents attending the local market (usually women and children, although men often work at markets or as roadside traders)	Once per week on average for individuals attending market, generally lasting under an hour

### Wider influences on air pollution exposure levels

In addition to quantitative findings, qualitative data demonstrated how scarcity shaped individuals’ smoke exposures throughout the village. These data could be summarised in three themes, described below: daily hardship, precarity and limitation.

#### Daily hardship

Village life for women involved daily physical and mental burdens. Women engendered the archetypal identity of a Malawian woman in this social setting through a daily resilience to these hardships. The difficulty of tending the fire and cooking *nsima* (thick maize meal, the staple food in Malawi) went beyond the smoky environment of the fire. Cooking involved a constant balancing of the heavy pot on the three support stones (*mafuwa*), while vigorously stirring the thick mixture, avoiding burns from spillages or extinguishing of the fire. We witnessed how proficiency in this important act was developed from childhood, with children helping their mothers and independently playing cooking games (*masanje*) involving real fires.

In addition to cooking activities, water was pumped from the local well and carried home in large buckets; clothes washed at the stream by pounding on rocks; and cooking pots vigorously rubbed (*kukwecha*) with sand and grit with the palm of the hand to remove black soot. These and other tasks—long walks to the market or maize mill for instance—were made more strenuous as they frequently took place under a hot sun, and were performed throughout pregnancies and while carrying babies in slings. Such daily hardships were recognised but rarely explicitly discussed by women themselves, for whom this just represented part of their normal lives.

Similarly, smoke itself was seen as unavoidable: a ‘fact of life’, as evidenced by the disbelieving response of, ‘utsi?’ (‘smoke?’) we became used to hearing on introducing the study topic in the village. There was no commonly used term for ‘air pollution’ in Chichewa, and the concept of air being polluted (in the way that drinking water might be) was not recognised by participants. Women did laughingly acknowledge the shared experiences of stinging eyes and running noses that we felt during cooking, but these were seen only as minor inconveniences. In conversation, attitudes were stoical:

Facilitator: (your eyes) they don’t hurt with the smoke?

Female Participant: they do hurt, so long as the nsima gets cooked, we just persevere

Workshop 3

On sharing knowledge of the longer term health effects of smoke exposure and our findings on ventilation, for example, some residents seemed concerned. However, we observed that these concerns quickly faded in the face of more immediate priorities. Throughout daily activities, an aggravating aspect was hunger. Residents themselves worked all year round on their farms to grow maize, the staple food source. Money to purchase small amounts of additional ingredients for daily *ndiwo* (stew eaten with *nsima*) was severely restricted. Meals mainly consisted of *nsima*, with small amounts of *ndiwo*, containing green vegetables and sometimes a protein source (beans, eggs, or dried fish). Sufficient food was not always available: at times the main meals constituted black tea and bread, and sometimes were missed entirely.

Sometimes we can eat in the morning, sometimes we don’t have food in the morning, so we wait for 12 o’clock, and then we also eat sometimes in the evening, so when we eat 3 times a day it depends on the availability of food.

Interview—female household head, household 3

Women did their utmost to manage food scarcity, striving to provide a respectable offering at every mealtime. Most women aspired to owning ‘kitchens’ (small standalone buildings) for cooking. One reason for this, although rarely explicitly mentioned, was the privacy they endowed: although economic scarcity was present throughout the village, participants did not wish this scarcity to be on public display.

…by our culture, women need privacy when cooking. That’s the reason why I don’t like cooking on the veranda. At times we may not have enough food stuff like tomatoes, onions, but you can still cook and eat what you can without people noticing.

Interview - female household head, household 6

Efforts to manage and safeguard oneself and family thus exacerbated the daily physical hardships witnessed in all households.

#### Precarity

Precarity refers to a state of precariousness or insecurity: a lack of stability. In the village, residents relied heavily on the crop harvest. A poor harvest, accompanied by food price increases, could mean protracted periods of hunger. Regular employment was rare, and reliance on temporary piece work deepened economic insecurity and increased the threat of sudden impoverishment. For women, the need to support a family accentuated this. Payments from male partners employed elsewhere were inconsistent. When initial funds for investment were available, women supplemented household income by microbusiness ventures, for example, roasting peanuts or bagging and reselling charcoal.

Climate factors exacerbated these daily insecurities. We witnessed floods which washed away a participants’ newly built kitchen, representing months of investment of time and money, and great disappointment among the (female) household head and the researchers. There was a widespread disinclination for longer term planning or saving among residents, which appeared a natural response to this climate of constant uncertainty.

Lack of motivation towards investment in long-term health or environmental improvement was evident. This included dismissive responses (laughter; ‘we’re busy’; ‘people can’t do that’) to our suggestions of tree planting, composting to replace burning of fields, or collective action to access cleaner water when the water from the local pumps appeared brown in the mornings.

#### Limitations on ‘choice’

**Figure 2:**
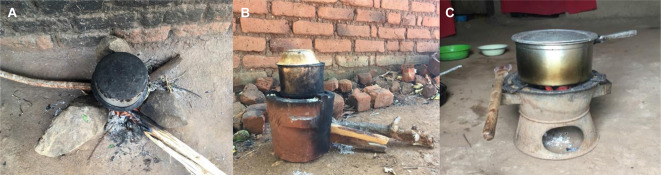
(A) Three stone fire, (B) firewood cookstove—‘chitetezo mbaula’, (C) charcoal cookstove.

Throughout participant observations, we saw how scarcity directly restricted individuals’ options, although this was at times complicated by gendered and culturally shaped choices. Use of three stone fires (figure 2A) for cooking, or eating of *nsima* as a daily staple, were often presented as pillars of Malawian culture, and as active choices. However, the landscape of these choices was constricted by availability and need. *Nsima* (made using only maize-flour and water) provided the most satiety for the lowest cost of any foodstuff. This was tacitly acknowledged by women who—in conversation about the contrast with typical British diets—stated that they would not be able to work long periods on the farm with ‘only bread’ in their stomachs.

For household cooking, three stone fires were the most frequently used by far, representing a ‘default’ method and often used even where alternative (firewood or charcoal) cookstoves were available. The fire was a traditional mode of cooking, familiar throughout life, to which people would revert when under pressure.

In normal circumstances we use firewood and that’s our culture

Interview—female household head, household 2

Interviewer: …but why do you still use the three stone fire most of the time?

Respondent: Because we are used to it.

Interview—female household head, household 5

Other motivations for using the three stone fire included the adaptability of the fires when dry firewood was scarce. At these times, a range of alternative substances were burned as fuel. Maize cobs and husks were used at harvest time, producing large amounts of smoke and burning for short periods, and roofing materials were sometimes burned, with residents replacing their roofs when money allowed. When times were particularly hard, bamboo mats and even household litter including clothes and shoes were burned for cooking fuel, as described by one participant:

I had to use my old reed mat to cook for my husband and child. I lit the fire with the pieces of the reed mat using the firewood stove. The whole house was filled with a huge mass of smoke. But I had no option but to cook for my family

Interview—female household head, household 6

Firewood cookstoves had been provided to some residents by government or non-governmental organization initiatives, but were regularly used in few households (*‘chitetezo mbaula’*, meaning ‘protective stove’ in Chichewa—shown in figure 2B). There was a visibly awkward physicality seen in women tending these stoves, with none of the easy expertise we had grown accustomed to seeing when women cooked on the three stone fire.

While the influences of custom and habit were apparent, the relatively restrictive fuel requirements for *chitetezo mbaula* were limiting. Charcoal cookstoves (figure 2C) were more widely owned, and particularly useful during the rainy season when firewood was damp. These mobile stoves could be used indoors to limit cooking disruption. Women often acknowledged improvements in smoke-related symptoms when cooking on charcoal:

…there is no smoke, there is nothing like you will be failing to breathe, no (Interview—female household head, household 1)

In these circumstances however, free fuel—firewood—was the preferred option for most. Charcoal stoves were used sporadically: charcoal was usually bought in small bags and used sparingly. Firewood scarcity was keenly felt by residents, who talked of the loss of trees in and around the village. Participants reported a desperation for fuel, leading some to fell trees around their households which had been important in providing shade, and even certain respected trees with purported medicinal properties. The occasional felling of trees around the village graveyard and breaking traditional taboos showed how immediate need compromised deeply held principles.

The theme of scarcity-related ‘limitation’, or ‘restriction’, returned in workshops as participants considered ways of reducing cooking-related smoke exposures. Suggested technological solutions were severely limited in their capacity to bring about real change as only the most basic of these would be financially feasible. Simple parabolic solar cookers, for example, could not be used for cooking *nsima* (due to the high power-output required) which, together with constraints relating to hours of sunlight, rendered them practically useless in this setting. Any stove which required purchased fuel was similarly impractical, as was cooking with electricity, to which residents commonly aspired.

The suggestion of ‘business’—described as development of an income-generating venture, allowing access to improved cooking methods—received a lot of support from the group. This highlighted participants’ awareness of the role of scarcity in framing smoke exposure in their lives.

### Combination of factors

The three themes above interacted in complex ways to shape individuals’ access to clean air. Even when residents came to know of the health impacts of smoke, limitations in terms of access to clean fuels and technologies, and competing priorities such as physical work, and securing an income and food for the day precluded any serious, sustained efforts to improve air quality.

## Discussion

This research demonstrates high levels of particulate air pollution throughout the rural Malawian village and the importance of cooking as a prominent source. Most residents were initially unaware of the impacts of smoke on health, and in fact did not conceptualise smoke in terms of ‘pollution’, or contamination. Through our time in the village, as residents became used to seeing us and familiar with our project, knowledge of these health effects became more widespread. Even so, our findings reveal how reduction of these exposures might require more than health education.

Simple cooking-related factors and gendered cultural norms in the setting contributed to individual exposures—such as in the use of three stone fires—but these factors in turn were powerfully constrained by overwhelming economic scarcity. Scarcity mediated individuals’ relationships with smoke through three mechanisms: limitations on choice, day to day hardships, and an underlying sense of precarity. These findings lead us to reframe ‘air pollution’ as one element of a wider system which structurally compromises health, and thus cannot be effectively managed in isolation.

Individual exposures to airborne particulate matter breached international standards even without cooking episodes, reinforcing other accounts,^6 78 79^ and reflecting the potential for adverse health effects.^4 77^ Cooking and other combustion sources may also contribute to the background or ambient air quality, as could environmental dust in this setting.^80^ Superimposed cooking-related exposures for women were particularly high in our analysis.

Evidence for the use of improved cooking technologies references themes of technology access and affordability,^30–34^ and describes clear relationships between socioeconomic status and technology uptake.^29 81^ Ethnographic evidence from the present study interrogates this relationship, considering the realities of life on the ground for women in rural Malawi, and their global origins.

Daily limitations were felt particularly by women, whose roles included ensuring the smooth running of their household and providing for all its members. We witnessed how ‘choices’ of cooking devices, fuels, place of cooking and even daily food were severely restricted by lack of access. This makes the extent of cultural influences on these practices unclear. An example of this was the use of three stone fires, where charcoal was in any case prohibitively expensive for most. Had there been a range of alternatives—if gas and electric stoves were freely available for instance—how then would these choices be made, and what would be the role of culture?

Apart from direct limitations on choice, individual experience of daily hardship and insecurity also shaped air pollution exposures in the village. Even when the health impacts of chronic smoke exposure were recognised, unpredictability and daily hardship left little room for women to consider smoke levels, still less to try to reduce smoke exposure. This echoes empirical observations that scarcity—defined simply as ‘having less’—impacts attention and decision making typically leading individuals to focus on immediate concerns at the expense of longer term high-level planning.^82 83^ Daniel Nettle proposes that we consider socioeconomic gradients seen in health behaviours across a population from the following preposition: ‘to the extent you see unpredictable health outcomes besetting your peers, worry about today rather than tomorrow’ (84, p.4). Nettle’s models suggest that actions to improve health follow an inverse U-shaped curve: there is an optimum amount of health behaviour, beyond which negative effects become apparent through their impacts on other aspects of life. Changes in levels of extrinsic mortality—that which cannot be mitigated by individuals’ health behaviours—affect these optimum amounts. In situations of high extrinsic mortality, such as those in the Malawian village, optimal amounts of health behaviour are low. Addressing extrinsic mortality through reductions in scarcity and insecurity may be a necessary precursor to positive ‘health behaviours’ such as changes to cooking.

Our approach to understanding air pollution in the village incorporates an examination of the origins of current inequities, described as ‘geographically broad’ and ‘historically deep’.^85^ This implicates colonialism, and subsequent extractive models of international relations. The structural adjustment programme, and subsequent debt dependence, has particularly impacted subsistence-reliant rural communities.^56^ This ethnography depicts the multiple ways in which systems of global inequity affect the experiences and choices of individuals in the Malawian village, shaping air pollution exposures through their lives.

Extreme climate events such as droughts and floods accentuated individuals’ precarity in the village setting. Such climate events are exacerbated by emissions of the most affluent global actors.^62^ Environmental colonialism hence leads to additional forms of structural violence.^86^ We witnessed a population dependent on biomass for cooking bound into complicity in local environmental degradation, thus worsening the negative impacts of serious climate events. On environmental destruction, Nixon’s theory of ‘slow violence’ builds on the structural violence concept, suggesting that the long timeframes over which environmental destruction occurs further obscures its origins.^87^ This makes restitution, redress, or prevention even harder to achieve.

This project combined fine-grain data on air quality with insights into individuals’ lived experiences in the rural Malawian setting. Our approaches counter the ‘decontextualising’ gaze which can be a feature of global health research efforts. Without recognising the powerful structural forces acting on individual and populations, recommendations relating to education and empowerment can be abstract and limited in their efficacy. Attention to broader context can help in considering effective responses to these complex population health issues.

Limitations relating to air quality sampling in our study may affect generalisability across the wider community. We did not capture certain activities, for example, men involved in burning brick ovens, so data on these areas are unavailable. A forthcoming manuscript reporting results from an extended, standardised dataset provides more extensive quantitative data. The monitors held in waist bags, although close to the face when cooks were in typical squatting position during cooking, could still underestimate inhaled particulate levels due to their lower position. If true, this adds gravity to the findings of particulate matter levels exceeding safe thresholds throughout.

Wider limitations include the necessarily context-specific nature of the ethnography, and our restriction to individuals present in the village. Community members living elsewhere, particularly men, may have differing perspectives to contribute, inclusion of which would be important in forming a more comprehensive account.

## Conclusion

This ethnography represents an in-depth, contextualised account of air pollution in a rural setting in Southern Africa. The results reveal how structural inequities, rooted in historical transnational relations, shape health concerns. Clean fuels for cooking—critical in bringing air pollution exposures in line with international standards—are currently inaccessible for rural Malawian communities in view of the associated costs and infrastructure, on both individual and governmental levels.^88^ Ultimately, complex global health issues such as air pollution demand broad, transdisciplinary approaches, placing communities and their experiences at the centre of research efforts. Solutions to these issues extend into the political sphere.^89^

## Data Availability

Data are available upon request. Due to potentially identifiable participant information, even where deanonymised, qualitative data are not publicly available but may be made available on individual request.
