# Supplementary material for: Exploring smoke: an ethnographic study of air pollution in rural Malawi
Source: BMJ Glob Health. 2021 Jun 30;6(6):e004970. doi: 10.1136/bmjgh-2021-004970 (PMC8246283; doi:10.1136/bmjgh-2021-004970)
Supplement: Supplementary data [file bmjgh-2021-004970supp001.pdf]

Supplementary materials

Approach

Throughout the project, I (SS) -- as a researcher from a British background -- maintained an awareness of potential pre-existing power differentials in the field, in terms of economic inequalities and the deeper imperialist contexts, and of their impacts on ethical research conduct and experiences in the field (1). A commitment to countering these imbalances started from a place of continuous reflection, ethical provisions, and a commitment to incorporating contextualised voices and perspectives as a driving force throughout the project (2). This in keeping with the wider epistemological approach of the project, moving beyond binaries of knowledge and power (that of intervention 'donor' and 'recipient' for instance) (3). The resulting ethnography could thus be likened to 'bricolage': an amalgamation of diverse knowledges around the issue of air pollution in the given context (4). While this approach has been important throughout our fieldwork and beyond, we, as researchers, also recognise the inherent limit to the redistribution of power in the project, given the central role of researcher in the development, implementation, and data assimilation into the final ethnographic product (5). Indeed, even the participatory elements of this project make no claims to true participatory action research.

Our initial introductions within the community were in keeping with the epistemological approaches outlined. Explanations around the rationale for the project involved conveying what we knew about the effects of 'smoke' on health, and explaining our desire to understand more about smoke in the village, aiming to work together to explore whether and how it might be possible to reduce smoke exposures in the village context. At all points through the project, we combined this open sharing of our knowledge with an expressed desire to learn from residents. This knowledge-seeking was evident in my continuing efforts to learn the language, which extended through all my interactions in the village, as well as an eagerness to 'learn through doing' in all aspects of daily life, including farming, bringing water and cooking activities.

This aspiration to a balanced sharing of knowledges, and a redressing of power differentials continued in our analysis. An example relates to our insights – through the extended period of engagement in the field – into the interplay between 'cultural practices' (such as cooking on a three stone fire) and elements of structural limitation affecting the availability of alternative choices. This apparent conflict relates to a potential critique of 'cultural relativism': broadly, the concept of understanding values and practices of a cultural group on its own terms, rather than judging from external perspectives (6). Farmer describes how cultural relativist approaches can sometimes conceal power differentials and injustice, through the 'othering' of ethnographic subjects (7).

In relation to cooking practices, while most women were accustomed to cooking on three stone fires, we saw how these practices and preferences were formed within a context of great limitation. Whilst our structural violence lens could be blamed for making disempowered 'victims' of participants in this context, we felt it important to acknowledge the limitations, and consider what (participants) might aspire to in their absence, rather than accepting the current situation as 'culturally normal'. This relates back to our critical approaches (8), interrogating contextual and historical factors underlying power differentials which shape current landscapes. Such approaches bring the possibility of a deeper, more equitable research engagement, representing empowerment and a liberation from limitations rather than their acceptance.

Methods

Participant observation

The initial participant observation elements of the study began with the researchers (the main researcher, SS, and research assistant, HS) walking around the village, discussing research plans with interested residents and hearing their views. Community introductions, including discussions with key figures in the community such as the chief and village health volunteer, were also part of this early work. The main part of the participant observation element – so-called 'focused household participant observation' – involved two researchers spending long days with household members, spending periods of 3 to 4 weeks at one household before moving to the next. In this time researchers lived, worked and rested alongside household members, taking part in activities including water collection, cooking, and farming, as well as accompanying residents on local outings, for example to the market.

Time spent with household members varied from long days, including the occasional night, to more focused time periods, incorporating meal preparation and eating, or farming activities for example. This developed into a form of theoretical sampling, taking place iteratively through the fieldwork period in response to changing relationships and findings in the field. Written field notes made during activities included observations around factors directly relating to smoke exposure, and also wider aspects of daily life which shaped these exposures, and ad hoc conversations in the field helped to probe more deeply into areas of interest.

Initial participant observations were carried out by the main researcher and research assistant but two months into the fieldwork period, a local resident joined the study team in the role of fieldworker. For subsequent participant observations the main researcher was accompanied by one or both of the research assistant and fieldworker. This afforded additional ethnographic perspectives, more akin those of an 'insider', given the fieldworker's residence in the village since birth and extensive familial connections within the community.

Village-based participant observation constituted the core of the ethnographic work, chosen in view of the contextualised nature of smoke exposures in the village setting, with the village itself constituting a 'culture-sharing group' (9). The household in particular has a central role in shaping the experiences of many people living in a rural setting such as Malawi. Starting at the level of individual households created a sort of graded entry into village life, helping us to become better known amongst the wider village community, allowing access to shared community sites and events, and enabling later spontaneous conversations throughout the village. These conversations generated continuing engagement throughout the project between researchers and residents, from early discussion of the research plans and recruitment, throughout subsequent project development.

In the weeks following completion of the six participatory workshops, the discussions with residents of the village as outlined above continued, now creating opportunities for wider engagement with the developing intervention ideas. Written fieldnotes from these discussions were analysed together with the participant observation data.

Limitations in the extent of involvement of participants in theme development and 'checking' were inherent in the study, however. Themes were inductively derived from daily experiences with residents, and collaboratively developed by research team members, allowing for some triangulation, and key ideas were broached for discussion at various points in the ethnographic period for discussion with residents. The nature of some of the latent themes – 'precarity', for example – informed by 'outsider' perspectives and western academic epistemologies, meant that analytical discussions of these topics with participants were often unproductive.

Individual interviews

At the end of each focused household period, an individual interview was arranged with a key member of the household. The purpose of this was to create space to recap some of the key findings and discuss potentially differing perspectives of researchers and household members, as well as to allow for deeper discussion of particular areas or issues where necessary. Interviews, carried out by the research assistant but with the main researcher also contributing, took place in Chichewa with the research assistant translating responses for the main researcher where necessary. Interviews were audio recorded and later translated into English and transcribed by the research assistant, with a Malawian transcriber fulfilling this role for a few of the later interviews for reasons of time. In these cases, completed transcripts were reviewed by the researcher and research assistant alongside recordings to ensure quality and consistency of transcription and translation. Although a denaturalised approach to transcription is not relevant where translation is also incorporated, both research assistant and transcriber used adapted denaturalised approaches where possible, to optimally represent the nature of the spoken conversation as it took place (10).

Air quality monitoring

During the later stages of household participant observation personal air quality monitoring was introduced, to provide quantitative information on exposures to airborne particulate matter by time, place, person, and activity. This component involved researchers carrying mobile air quality monitors in small waist bags alongside participant observations. At times a small number of household members were asked to continue carrying monitors (in waist bags) overnight after researchers left the household, with a short 'debrief' the following morning when the monitor was returned, to register key potential exposure points. This quantitative data lies outside the scope of the current report – the outlining of the methods above provided only for completeness.

Participatory work

A series of six once-weekly participatory workshops conducted in Chichewa, involving approximately 15 participants (members of the local community) and the three field researchers, were arranged alongside the final weeks of participant observation. These workshops aimed to further explore the different sources of smoke in participants' daily lives and to allow people to think together about ways of reducing their levels of smoke exposure. Workshops were led by an external consultant (EM): a community theatre practitioner and researcher who, whilst British by background, has many years of experience working with communities in Malawi and whose approaches fit closely with the epistemologies underlying the project. EM specialises in the use of participatory theatre methods for research purposes, in particular Boal's Theatre of the Oppressed (11), using elements of physicality and the body to break down conventional 'researcher/researched' dynamics, and an action-reflection discourse through the processes to explore realities as they are, and to co-create imagined futures. This action-oriented research approach was well-suited to the current project, creating a community of co-learning and questioning, providing valuable spaces for researchers and participants to exist as individuals together, and opportunities to uncover new knowledges that might otherwise be harder to access (12). The parallel involvement of research team members and residents allowed this to act as an additional component of the ethnography. Five of the six workshops were audio recorded (the first workshop, being active in nature and involving mainly familiarisation games, was not).

Results

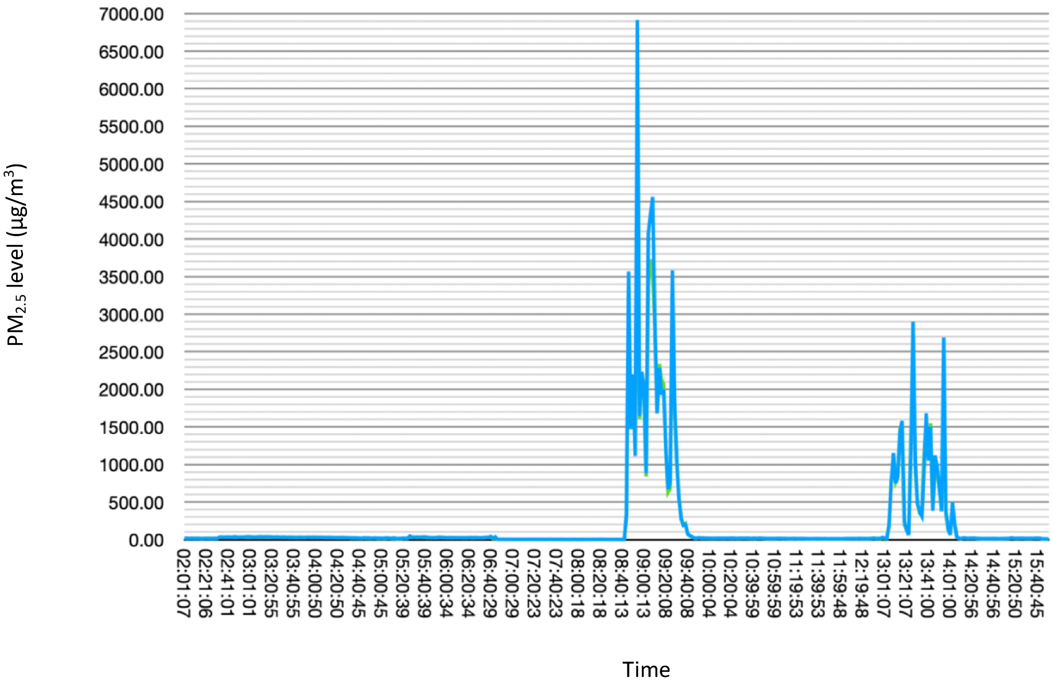

Supplementary Figure S1. Sample $PM_{2.5}$ trace for a female household resident

		Coefficient	Standard Error	P value	95% Confidence Interval	
Activity	No activity					
	Cooking	291.7	90.4	0.001	114.5	468.9
	Other	60.0	57.7	0.298	-53.0	173.0
Stove	NA					
	Three stone	-234.0	233.9	0.317	-692.4	224.4
	Mbaula firewood	-654.2	239.6	0.006	-1123.7	-184.6
	Mbaula charcoal	210.8	133.5	0.114	-50.8	472.5
Location	NA					
	Walled veranda	200.8	36.4	<0.001	129.4	272.2
	Unwalled veranda	-164.2	41.1	<0.001	-244.8	-83.6
	Outdoors	-133.5	54.9	0.015	-241.1	-25.9
Fuel	Firewood	639.0	235.6	0.007	177.3	1100.7
	Constant	104.7	55.1	0.058	-3.3	212.7

Random-effects parameters	Estimate	Standard Error	95% Confidence Interval	
ID: Identity				
sd (_cons)	302.4	43.0	228.9	399.6
sd (residual)	443.7	4.0	435.9	451.7

Supplementary Table S1: Mixed level regression model to determine the relationship between individual and activity on mean $PM_{2.5}$ exposure. There were 6091 individual observations with 31 groups. The minimum observations per group were 27 and maximum 596 (average 197). Wald chi^2 = 2904.35

References

1. Fechter A. Cultures in the Classroom: Teaching Anthropology as a "Foreigner" in the UK. Anthropology Matters Journal [Internet]. 2003 [cited 2020 23 June]; 5(1). Available from: https://www.anthropologymatters.com/index.php/anth_matters/article/download/128/252?inline=1.
2. Biehl J. Theorizing global health. Medicine Anthropology Theory. 2016;3:127-42.
3. Brydon D. modes and models of postcolonial cross-disciplinarity. 2013 [cited 20 June 2020]. In: The Oxford Handbook of Postcolonial Studies [Internet]. Oxford University Press, [cited 20 June 2020]. Available from: https://www.oxfordhandbooks.com/view/10.1093/oxfordhb/9780199588251.001.0001/oxfordhb-9780199588251-e-016.
4. Denzin NK, Lincoln YS. Handbook of Qualitative Research: SAGE Publications; 1994.
5. Stacey J. Can there be a feminist ethnography? Women's Studies International Forum. 1988;11(1):21-7.
6. Tilley JJ. Cultural Relativism. In: Ritzer G, editor. Wiley-Blackwell Encyclopedia of Sociology, 2nd ed: Wiley-Blackwell.
7. Farmer P. Pathologies of Power: Health, Human Rights, and the New War on the Poor: University of California Press; 2004.
8. Farmer P. An Anthropology of Structural Violence. Current Anthropology. 2004;45(3):305-25.
9. Guetterman TC. Descriptions of Sampling Practices Within Five Approaches to Qualitative Research in Education and the Health Sciences. 2015. 2015;16(2).
10. Bucholtz M. The politics of transcription. Journal of Pragmatics. 2000;32(10):1439-65.
11. Boal A. Theater of the Oppressed. Boston: Theater Communications Group; 1985 (1974).
12. Etmanski C. ‘Theatre of the Oppressed’. The SAGE Encyclopedia of Action Research. California: SAGE Publications Ltd; 2014.
